# A striking new species of *Dioon* (Zamiaceae) from pine and pine-oak forest of Guerrero, Mexico

**DOI:** 10.3897/phytokeys.274.173907

**Published:** 2026-05-11

**Authors:** Lilí Martínez-Domínguez, Fernando Nicolalde-Morejón, Dennis Wm. Stevenson, Francisco G. Lorea-Hernández, Francisco Vergara-Silva

**Affiliations:** 1 Programa de Posgrado en Botánica, Colegio de Postgraduados, Km. 36.5 carretera México-Texcoco, Montecillo 56264, Estado de México, Mexico Programa de Posgrado en Botánica, Colegio de Postgraduados Montecillo Mexico; 2 Laboratorio de Teoría Evolutiva e Historia de la Ciencia (Jardín Botánico), Instituto de Biología, Universidad Nacional Autónoma de México, 3er. Circuito Exterior, Ciudad Universitaria, 04510 Coyoacán, Ciudad de México, Mexico Universidad Nacional Autónoma de México Ciudad de México Mexico; 3 Laboratorio de Taxonomía Integrativa, Instituto de Investigaciones Biológicas, Universidad Veracruzana, 91190, Xalapa, Veracruz, Mexico Universidad Veracruzana Xalapa Mexico; 4 The New York Botanical Garden, Bronx, New York 10458-5120, USA The New York Botanical Garden New York United States of America; 5 Red de Biodiversidad y Sistemática, Instituto de Ecología, A.C., Xalapa, 91073, Veracruz. Mexico Red de Biodiversidad y Sistemática, Instituto de Ecología Xalapa Mexico

**Keywords:** Cycadales, cycads, Mesoamerica, Neotropics, Sierra Madre del Sur

## Abstract

Taxonomic studies in cycad genera using multiple approaches have refined the delimitation of many species. In the case of *Dioon* Lindl., a Mesoamerican genus, a reliable classification has been achieved through taxonomic work carried out since the description of the genus and pioneering studies in Mexico during the 1980–90s. Here, we describe a new species from Guerrero based on evidence collected from populations encountered during fieldwork carried out in 2019. These populations had previously been considered morphologically similar to *Dioon holmgrenii* De Luca, Sabato & Vázq. Torres, which has a markedly disjunct distribution in Oaxaca. After studying herbarium specimens and making extensive observations on vegetative and reproductive structures from different populations in Oaxaca, we have concluded that the disjunct populations analysed in 2019 represent a distinct and new species of *Dioon*, separate from the two most phenotypically similar species—namely, *D. stevensonii* Nic.-Mor. & Vovides and *D. holmgrenii*. A key to geographically proximal and morphologically similar species as well as to the other species occurring in Guerrero and Oaxaca States is also presented. The proposed new species, *Dioon nuusaviorum* Mart.-Domínguez, Nic.-Mor. & D.W.Stev., is endemic to Guerrero and inhabits pine and pine-oak forest. Its conservation status, assessed on the based IUCN guidelines and criteria, qualifies as Endangered.

## Introduction

*Dioon* Lindl., one of the three cycad genera (*Ceratozamia* Brongn., *Dioon* and *Zamia* L.) present in Mexico, comprises 18 species worldwide (Nicolalde-Morejón et al. 2014; Calonje et al. 2025). Phylogenetic and phylogeographic analyses based on nuclear and chloroplast DNA sequences support the monophyly of *Dioon* (González et al. 2008; Dorsey et al. 2018; Gutiérrez-Ortega et al. 2018b); phylogenetic relationships within the genus are highly resolved and relatively well supported. Additional phylogeographic studies using single nucleotide polymorphisms proposed orogeny and aridification as important factors in the speciation of *Dioon* (Gutiérrez-Ortega et al. 2018a).

The genus has a disjunct distribution between Mexico and Honduras, occurring along the mountains of Sierra Madre Oriental, Sierra Madre Occidental, and Sierra Madre del Sur (Dorsey et al. 2018; Gutiérrez-Ortega et al. 2018a). The circumscription of species within the genus has changed over time as taxonomic concepts have been refined (Haynes 2020; Hernández-Tapia et al. 2020; Martínez-Domínguez et al. 2024a). The largest number of new taxa in *Dioon* were published between 1980 and 1990, when about 30% of the currently recognized species were described (Martínez-Domínguez et al. 2024a). Since the most recent taxonomic treatment of *Dioon* (Hernández-Tapia et al. 2020), two additional species from Mexico have been described (Calonje et al. 2025). These two species, *D. salas-moralesiae* Gut.Ortega & Pérez-Farr. and *D. oaxacensis* Gut.Ortega, Pérez-Farr. & Vovides, were described from populations previously assigned to *D. merolae* De Luca, Sabato & Vázq.Torres (Gutiérrez-Ortega et al. 2020a, 2020b).

Most *Dioon* species have restricted geographic distributions, including *D. angustifolium* Miq. (from Nuevo León to southern Tamaulipas), *D. argenteum* T.J.Greg., Chemnick, Salas-Mor. & Vovides (endemic to northern Oaxaca), *D. califanoi* De Luca & Sabato and *D. caputoi* De Luca, Sabato & Vázq.Torres (both occurring at the boundaries between southern Puebla and northern Oaxaca), *D. oaxacensis* (endemic to Oaxaca), *D. planifolium* Salas-Mor., Chemnick & T.J.Greg. (endemic to northwest Oaxaca), *D. purpusii* Rose (endemic to Oaxaca), *D. rzedowskii* De Luca, A.Moretti, Sabato & Vázq.Torres (endemic to La Cañada region in Oaxaca), *D. salas-moralesiae* (endemic to the Isthmus region of Oaxaca), and *D. vovidesii* Gut.Ortega & Pérez-Farr. (endemic to Sonora; Calonje et al. 2025). In contrast, several other species such as *D. mejiae* Standl. & L.O.Williams (widely distributed in Honduras), *D. holmgrenii* (western portion of the Sierra Madre del Sur province in Oaxaca), *D. merolae* (Chiapas and Oaxaca), *D. spinulosum* Dyer ex Eichler (distributed in the northwestern Oaxaca), *D. stevensonii* Nic.-Mor. & Vovides (Michoacán to northwest Guerrero), *D. tomaselli* De Luca, Sabato & Vázq.Torres, and *D. edule* Lindl. are comprised of several populations (Nicolalde-Morejón et al. 2014). In particular, the latter two are the most broadly distributed in terms of latitude and elevation (Hernández-Tapia et al. 2020). *Dioon edule* occurs in southwestern Mexico and in the mountains of San Luis Potosí, Querétaro, Hidalgo, Veracruz and Tamaulipas, from sea level up to 1,200 m. In turn, *D. tomaselli* occurs in Durango, Jalisco, Nayarit and Sinaloa States from 600 m up to 1,900 m. Both *D. edule* and *D. tomaselli* inhabit several vegetation types including tropical deciduous forest, pine-oak forest, pine forest, oak forest, and tropical rain forest.

Allopatric speciation driven by niche conservatism has promoted lineage divergence, and consequently, high cycad species diversity, particularly in *Dioon* and *Ceratozamia* (Gutiérrez-Ortega et al. 2020a; Habib et al. 2023; Martínez-Domínguez et al. 2024b). The Sierra Madre del Sur, with its heterogeneous topography and climate, harbors high cycad diversity, and several species have been described from the region. This biogeographic province is divided into subprovinces, within which two districts —the Oaxaca and the Guerrero highlands— form part of the eastern Sierra Madre del Sur subprovince (Morrone 2010). Oaxaca is the state with the highest species richness and is considered one of the centers of cycad diversity in Mesoamerica (Vovides et al. 2003). In contrast, Guerrero is among the least studied states in terms of cycad diversity. For example, *Ceratozamia leptoceras* Mart.-Domínguez, Nic.-Mor., D.W.Stev. & Lorea-Hern., was described only recently based on a specimen collected in 1984 that remained in some herbarium case unprocessed for decades (Martínez-Domínguez et al. 2020).

The current cycad diversity of Guerrero encompasses three species —namely *Ceratozamia leptoceras*, *Dioon stevensonii*, and *Zamia paucijuga* Wieland (Nicolalde-Morejón et al. 2014; Calonje et al. 2025). *Ceratozamia leptoceras* is endemic to cloud forest in Guerrero between 1,170 and 1,400 m, whereas the other two species are more widely distributed along the coastal Pacific region. *Zamia paucijuga* occurs from Nayarit to Oaxaca between sea level and 1,200 m in pine, tropical deciduous and subdeciduous forests, including secondary vegetation and cultivated areas. *Dioon stevensonii* extends from Guerrero into Michoacán, occurring in oak and tropical deciduous forests from 500 to 1,200 m (Nicolalde-Morejón et al. 2009, 2014).

The studies of several botanists who have worked in the Sierra Madre del Sur have produced comprehensive lists of endemic vascular plants (Acosta et al. 2003; Contreras-Medina 2016; Aragón-Parada et al. 2021). These authors have highlighted the diversity of vascular plants species associated with conifer and oak forests in the states of Jalisco, Oaxaca, and Guerrero (Aragón-Parada et al. 2021). Considering this data and our extensive examination of herbarium specimens, we can confidently state that there are no records of *Dioon* from the southeast of Guerrero.

During fieldwork in Guerrero in 2019, when we were studying populations of *Ceratozamia leptoceras* and describing its reproductive structures and phenology, we discovered hitherto unknown populations of *Dioon* that could not be assigned to any known species in the genus. In subsequent years, additional searches for new *Dioon* populations in Oaxaca were conducted to evaluate the identity of the Guerrero populations. A better understanding of population-level morphological variation across these localities provided the basis for proposing the corresponding Guerrero specimens as a new species.

In this work, our objective has been to corroborate or refute a hypothetical species from southwest Guerrero. We have emphasized the analysis of both reproductive and vegetative phenotypic variation. We describe our results in the context of detailed morphological comparisons with all known species of *Dioon*, and provide illustrations, notes on morphology, a preliminary extinction risk assessment, and a taxonomic key to *Dioon* species in Guerrero and those morphologically close to the new species from Oaxaca.

## Material and methods

### Sample collections and character selection

**Figure 1.**
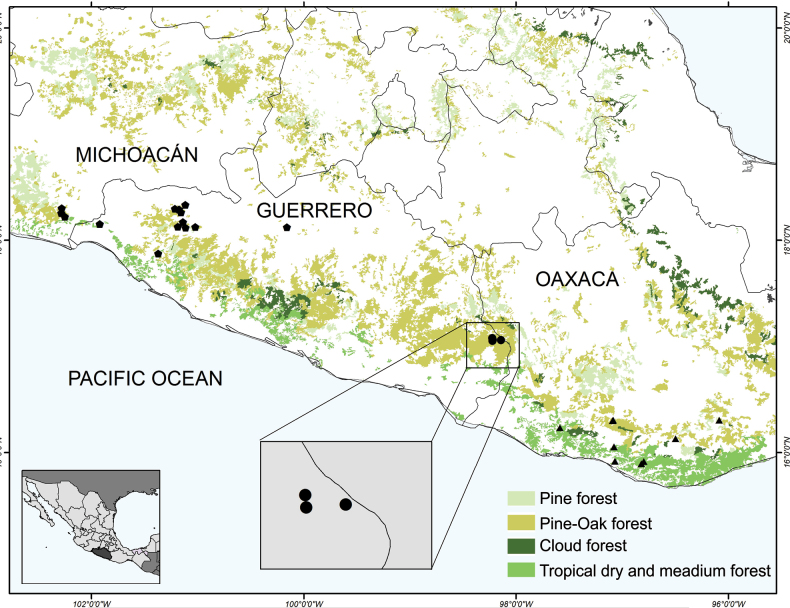
Distribution map of the species *Dioon stevensonii* (pentagons), *D. holmgrenii* (triangles), and *Dioon nuusaviorum* sp. nov. (dots). Inset: points corresponding to the new species.

Fieldwork was conducted in three populations in the Guerrero highlands of the Sierra Madre del Sur (Suppl. material 1: table SS1). We examined 10 to 15 individuals per population, recording both quantitative and qualitative morphological characters. Two morphological matrices were constructed using the data obtained in the field studies. The qualitative matrix has 24 characters, of which 9 are reproductive and 15 vegetative (Suppl. material 1: table SS2). The quantitative matrix has 15 reproductive and 22 vegetative characters (Suppl. material 1: table S3). The reproductive characters were measured directly in the field. Herbarium voucher specimens were deposited in the CIB and MEXU herbaria (herbarium acronyms from Thiers updated continuously). These specimens were compared to all previously known *Dioon* species, with a focus on those that are morphologically similar and geographically proximal, i.e., *D. stevensonii* and *D. holmgrenii* (Fig. 1). A taxonomic key is presented for these species and those morphologically close to the new species from adjacent Oaxaca —namely, *D. planifolium*, *D. salas-moralesiae*, *D. oaxacensis*, *D. purpusii*, *D. argenteum*, *D. caputoi* and *D. califanoi*. Type specimens of all these species were reviewed in detail.

### Geographic distributions

Specimens from the following herbaria were examined: CIB, CHAPA, K, MEXU, MO, NY, UAMIZ, SERO, and XAL (acronyms according to Thiers updated continuously). A distribution map was made using ArcMap 10.2 (Esri, Redlands, USA). Regional and vegetation types follow Morrone et al. (2022) and the most recent land use and vegetation coverage (series VII) from the Instituto Nacional de Geografía y Estadística (INEGI).

We evaluated a preliminary conservation status for the new species based on the IUCN Red List categories and criteria (IUCN 2025). The category was assessed using the Geospatial Conservation Assessment Tool (GeoCAT; Bachman et al. 2011).

### Qualitative and quantitative analyses

To explore morphological patterns and estimate diagnosability, we used the two qualitative morphological matrices (vegetative and reproductive) and appplied Population Agreggation Analysis (PAA; Davis and Nixon 1992). Characters fixed among populations were used to develop the diagnoses for the comparative table and the diagnosis of the new species. Quantitative vegetative characters were analyzed independently for the characterization of species (Suppl. material 1: table S3). Analysis of variance (ANOVA) and a Tukey Honest Significant Difference (HSD) test were performed to evaluate statistically significant mean patterns between population pairs. Also, a Principal Component Analysis (PCA) was used to characterize quantitative variation at the species level.

## Results

### Qualitative morphological characters

**Figure 2.**
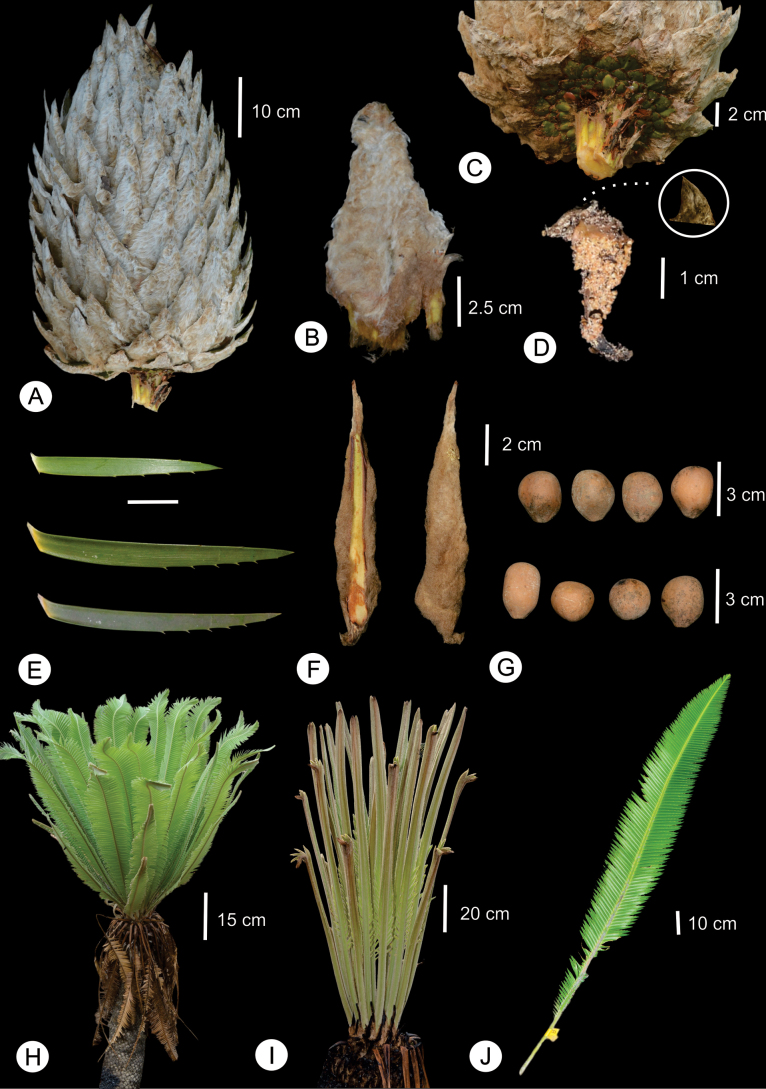
*Dioon nuusaviorum* sp. nov. **A**. Ovulate strobilus; **B**. Megasporophyll; **C**. Basal scale of megasporophyll; **D**. Microsporophyll; **E**. Leaflets variation; **F**. Cataphylls; **G**. Seeds variation; **H**. New leaves; **I**. Leaves at emergence; **J**. Leaf at maturity.

Vegetative morphological patterns were generally congruent among, and within, *Dioon* species. Fixed patterns of variation allowed clear diagnosability among the three morphologically similar species, *D. holmgrenii*, *D. stevensonii* and *Dioon nuusaviorum*. The most informative characters for the recognition of the three species were: (i) leaf color at emergence, (ii) leaflet orientation (on rachis), (iii) leaflet imbrication, and (iv) leaflet direction on margin (Fig. 2E). Qualitative vegetative morphological differences between *Dioon nuusaviorum* and the other two species were conspicuous because this species has imbricated and acroscopically curved leaflets (Table 1). Although additional characters were informative, these were difficult to distinguish in herbarium specimens (Table 1). In this context, *D. stevensonii* shares some morphological characters with the new species but differs by leaflets (particularly on the apical ones) with more and longer marginal teeth, and basal scales of light green to yellowish green megasporophylls with an acute apex.

**Table 1.** Comparison of both vegetative and reproductive diagnostic qualitative morphological characters.

Characters	*Dioon nuusaviorum* sp. nov.	*D. holmgrenii*	*D. stevensonii*
Leaf color at emergence	Light green	Light green	Golden brown
Trichomes on leaves at emergence	Light brown	Brown	Golden
Leaf orientation	Drooping	Ascending	Ascending
Leaflet shape	Linear-lanceolate	Linear-lanceolate	Linear-lanceolate
Leaflet imbrication	Imbricate to strongly imbricate	non-imbricate	non-imbricate
Leaflet margin orientation	Curved acroscopically	Straight	Straight
Megasporophylls shape	Narrowly-triangular	Narrowly-triangular	Triangular
Indument on megasporophylls at maturity	Tomentose	Tomentose	Pubescent at base or scarcely pubescent
Basal scales color of megasporophylls	Light green to yellowish green	Green	Dark green
Apex shape of basal scales megasporophylls	Acute	Apiculate	Apiculate
Apex shape of megasporophylls	Apiculate	Acuminate	Acute
Apex shape of microsporophylls	Acuminate	Acute	Acute

Reproductive morphological characters were the most useful for species identification because each of the three species exhibits a unique combination of character states (Table 2). *Dioon nuusaviorum* and *D. holmgrenii* have long triangular and tomentose megasporophylls, but the former has megasporophylls with an apiculate apex. In contrast, *Dioon stevensonii* has triangular and sparsely pubescent megasporophylls that may be glabrous and acute at the apex. Pollen strobili were broadly similar in these three species, except for the shape of the microsporophyll apex that is acuminate in the new species but acute in *D. holmgrenii* and *D. stevensonii* (Table 1).

**Table 2.** Comparison of diagnostic quantitative morphological characters, both vegetative and reproductive. Characters are in cm. * Meristic characters.

Characters	*Dioon nuusaviorum* sp. nov.	*D. holmgrenii*	*D. stevensonii*
Number of denticles on the leaflets*	3–6	2–5	1–3
Denticles length on the leaflets	0.26–0.37	0.15–0.29	0.01–0.1
Width of median leaflets	0.65–0.98	0.7–0.9	0.6–1.1
Length of median leaflets	6.8–12.3	7.2–12.5	7–14
Petiole length	12–18	13–30	8–15
Distance between median leaflets	0	0.2–1.0	0.3–0.5
Length of pollen strobili	42–44	40–43	20–24
Length of microsporophylls	3.4–3.8	1.6–3.0	2.9–3.8
Width of microsporophylls	1.5–2.0	2.0–2.4	1.1–1.8
Length of ovulate strobilus	45–56	30–50	30–35
Peduncle length of ovulate strobilus	6.0–7.2	1.8–4.5	3.0–5.0
Megasporophylls length	8.5–12	5–12	7–8.3
Megasporophylls width	4.8–5.4	2.8–4.5	4.5–6

### Characterization of quantitative morphological variation

Analysis of vegetative quantitative characters indicated that each species differs significantly from the other two morphologically similar species (Suppl. material 1: fig. S1, table S4). Likewise, a Tukey Honest Significant Difference showed that 12 quantitative characters have significant different mean values among the three species (Suppl. material 1: table S4). In pairwise comparisons, the most informative traits for distinguishing the species were the number and length of denticles, petiole length, distance between median leaflets, and width of the apical leaflets (Suppl. material 1: fig. S1, table S4).

Principal Component Analysis (PCA) components 1 and 2 together explained 56.5% of the total variation. The characters contributing most to these components were number of denticles, distance between median leaflets, number of leaflets, distance between basal leaflets, and length of apical leaflets (Suppl. material 1: fig. S2). Individuals of the new species tended to separate from *Dioon holmgrenii* and *D. stevensonii*, which were slightly more similar to each other.

### Species delimitation

A combination of geographic localization data, coupled with reproductive and vegetative morphology, has allowed us to propose the recognition of this new species from Guerrero. A unique combination of qualitative character states supports this new species. The quantitative evidence could be attributed to morphological plasticity; however, populations of the three species occur in similar environment and climatic conditions and show a strong pattern of species clustering in morphological space, in which no overlaps were detected. This pattern of character data therefore corroborates the species hypothesis for the populations from southeastern Guerrero.

### Taxonomic treatment

#### Dioon nuusaviorum

Taxon classification

Plantae

CycadalesZamiaceae

Mart.-Domínguez, Nic.-Mor. & D.W.Stev. sp. nov.

C4B21994-A252-5147-86F5-9A12AFB2D650

urn:lsid:ipni.org:names:77379798-1

Figs 2, 3, 4

##### Diagnosis.

*Dioon nuusaviorum* sp. nov. differs from *D. holmgrenii* by having leaflets imbricate to strongly imbricate, each with three to six long marginal teeth (0.26–0.37 cm long), a margin of the leaflets curved acroscopically, microsporophylls with an acuminate apex and megasporophylls with an apiculate apex. In contrast, *D. holmgrenii* has non-imbricate leaflets (generally a 0.2–1.0 cm between leaflets) with two to five short teeth on the distal margin (0.15–0.29 cm long), a margin of the leaflets straight, microsporophylls with an acute apex and megasporophylls with an acuminate apex. In comparison to *D. stevensonii*, this new species differs by its light green leaflets at emergence (vs golden), leaflet imbricate to strongly imbricate (vs not imbricate), acuminate apex of microsporophylls (vs acute), tomentose indument and narrowly-triangular megasporophylls at maturity (vs pubescent at base or scarcely pubescent and triangular).

**Figure 3.**
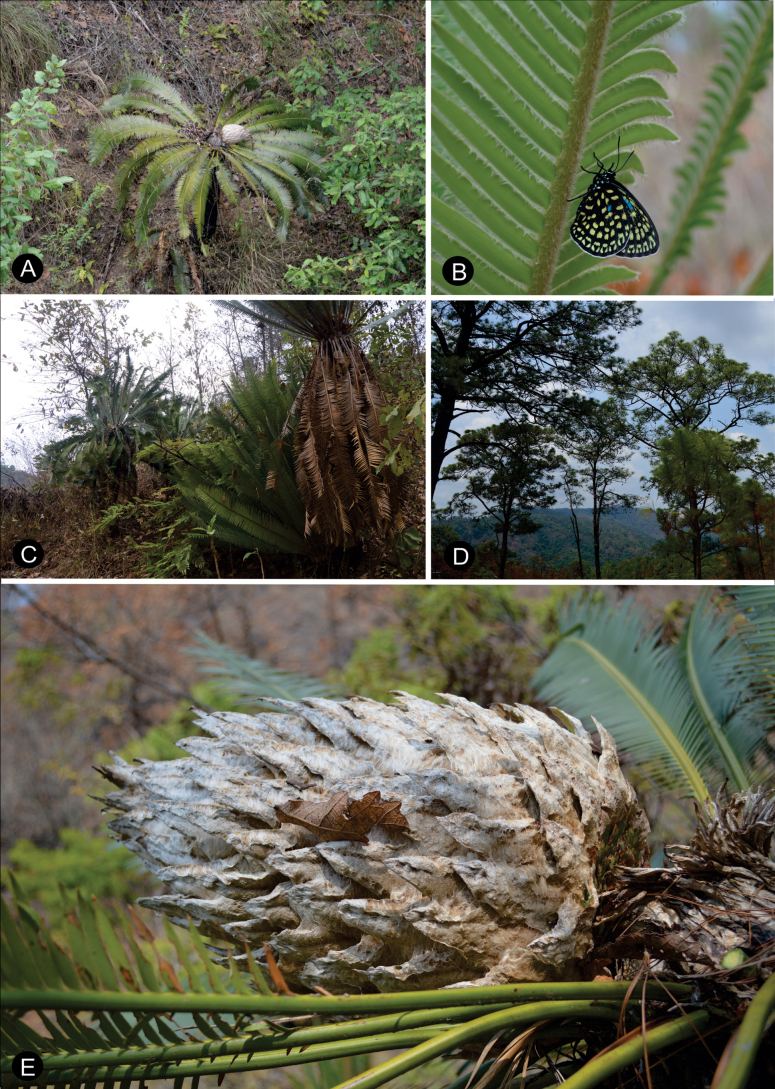
*Dioon nuusaviorum* sp. nov., in habitat. **A**. Ovuliferous plant in habitat; **B**. *Eumaeus* sp; **C**. Population in La Trinidad; **D**. Vegetation view; **E**. Ovulate strobilus at maturity in habitat.

**Figure 4.**
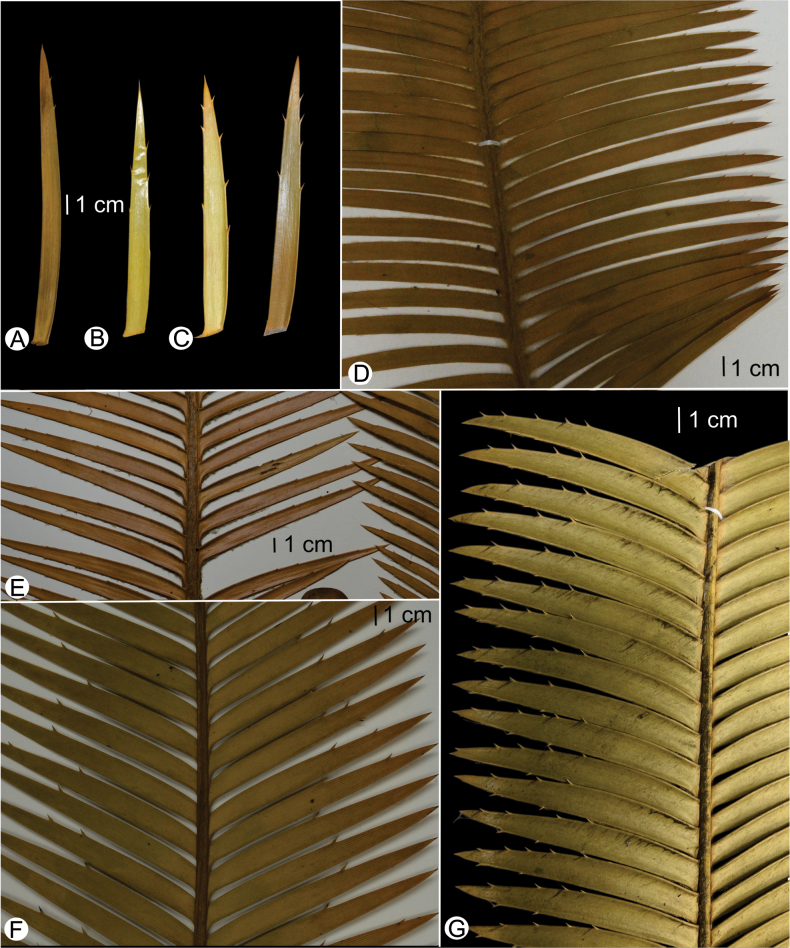
Comparison of leaflets of *Dioon holmgrenii*, *D. stevensonii* and *Dioon nuusaviorum* sp. nov. **A**. *D. stevensonii*; **B**. *D. holmgrenii*; **C**. *Dioon nuusaviorum* sp. nov; **D**. *D. stevensonii* (*F. Nicolalde-Morejón et al. 1554*, CIB); **E**. *D. holmgrenii* (*Brigada T. Walters s/n* [3997], XAL); **F**. *D. holmgrenii* (*F. Nicolalde-Morejón et al. 1468*, XAL); **G**. *Dioon nuusaviorum* sp. nov. (*L. Martínez-Domínguez et al. 1745*, CIB).

##### Type.

Mexico • Guerrero: Tlacoachistlahuaca, near Rancho Viejo, 1,140 m elev., 28 May 2019, *F. Nicolalde-Morejón et al. 3156* (holotype CIB, 22584UV; isotype MEXU).

##### Description.

Stem epigeous, erect to decumbent, up to 300 cm in length, up to 32 cm in diameter, covered by persistent leaf bases, sometimes bifurcate in mature plants. Cataphylls persistent, coriaceous, linear to narrowly triangular, yellowish green, densely light brown tomentose abaxially at emergence, densely pubescent at senescence, apex acuminate, 9.0–13.0 × 0.5–1.2 cm at base. Leaves 20–50, dropping at maturity, 100–150 cm long, light green at emergence, tomentum brown at emergence, turned grayish brown later, glabrous at maturity. Petiole subterete, 12–18 cm long, unarmed, green in mature leaves, densely light brown tomentose when young and glabrous at maturity. Rachis subterete, linear, 85–174 cm long, unarmed, green in mature leaves, densely light brown tomentose when young and glabrous at maturity. Leaflets 97–167 pairs, linear-lanceolate, imbricate to strongly imbricate, coriaceous, opposite to subopposite, acroscopically curved margin, plane, green, adaxial and abaxial surfaces glabrous, apex acuminate and non-reflexed, base attenuate, articulations yellowish, inserted at right angles on the rachis, margins deeply denticulate, with up to four serrations on the distal margin, and 1 or 2 on the proximal margin, denticles 0.26–0.37 cm long; median leaflets 6.8–12.3 × 0.65–0.98 cm long. Pollen strobilus solitary, ovoid when emerging, angular cylindrical at maturity, densely light brown tomentulose, 42–44 cm in length, 8.2–8.5 cm in diameter, light brown pubescent at emergence, greyish brown pubescent at maturity; peduncle tomentulose, brown, 5.0–7.2 cm in length, 2.0–3.0 cm in diameter; microsporophylls 3.4–3.8 × 1.5–2.0 cm, cuneiform, fertile portion covering 2/3 of the abaxial surface, sterile portion triangular, reflexed distally ending with a slight pungent apex, 1.23–1.3 cm long; synangia with 4–5 sporangia; 1.3 a 1.5 cm distal end infertile. Ovulate strobilus solitary, ovoid, erect, 45–56 cm in length, 25.46–27.5 cm in diameter, light brown tomentulose at emergence, greyish brown tomentulose at maturity; peduncle tomentose, brown, 6.0–7.2 cm in length, 3.5–4.2 cm in diameter, basal scales of megasporophylls light green to yellowish green with acute apex; megasporophylls 8.5–12 × 4.8–5.4 cm, strongly imbricate, distal portion triangular with an apiculate and non-reflexed apex, green, tomentose. Seeds ovoid, 2.77–3.35 cm in length, 1.99–2.31 cm in diameter, light brown at maturity, sarcotesta light yellow at maturity, sclerotesta smooth with 13 to 15 radial ridge-like markings extending from micropylar to chalazal end.

##### Additional specimens examined

**(*paratypes*)**. Mexico • Guerrero: Tlacoachistlahuaca, near Rancho Viejo, 1,190 m, 28 May 2019, *F. Nicolalde-Morejón et al. 3152-3155* (CIB, 22580UV; 22581UV; 22582UV; 22583UV); *L. Martínez-Domínguez et al. 1745* (CIB, 22079UV), 1746 (22080 UV, XAL); 1747-1749 (CIB, 22081UV; 22082UV; 22083UV); La Trinidad, 1,030 m, 28 May 2019, *F. Nicolalde-Morejón et al. 3157* (CIB, 22585UV), 3158 (XAL), 3159-3162 (CIB, 22587UV; 22588UV; 22589UV; 22590UV); *L. Martínez-Domínguez et al. 1750* (XAL), 1751-1755 (CIB, 22085UV; 22086UV; 22087UV; 22088UV; 22089UV).

##### Etymology.

“Ñuu savi” is an endonym translated as “people of the rain” (“Ñuu savi” has come to substitute “Mixtec” as the name of this ethnic group, as the latter is a Spanish adjustment of *mixtecatl*, the Nahuatl name for “cloud people”).

##### Distribution and ecology.

*Dioon nuusaviorum* is known only from the Sierra Madre del Sur, in the Guerrero highlands district of Mexico. The habitat is pine and pine-oak forest, with an elevation between 1,030 and 1,190 m, composed of clay soils with scattered karstic rocks. The region has temperate climate with a pronounced rainy season during the summer months.

##### Phenological observations.

Leaf production occurs annually in May. On average, each adult plant produces 12–18 leaves, although some individuals produce only 9–10 leaves. Ovulate strobili were observed in the late ovulate phenophase from May to June, and pollen strobili were recorded in the open pollen phenophase from March to April.

##### Preliminary conservation status.

Three populations of this new species were recorded in the study area (Fig. 1). They thrive in pine-oak and pine forests, which are highly threatened ecosystems due to frequent forest fires (Alfaro-Reyna et al. 2020). Each population has more than 50 adult individuals. Two of the populations are threatened because they occur in areas disturbed fire. In contrast, one population is in a less fire-prone site and appears to be in a better condition for conservation (Fig. 3C). Population sizes are relatively high, with good recruitment of seedlings and juvenile plants. In addition to fire, a major conservation threat to the species is the expansion of the agricultural frontier. Based on calculations from GeoCAT, the extent of occurrence (EOO) is 11.662 km^2^ and the area of occupancy (AOO) is 12.000 km^2^. Following the IUCN criteria, the species is preliminarily assessed as Endangered [EN (B1ab (ii, v)); C2a(i)].

### Taxonomic key to the *Dioon* species in Guerrero and those morphologically close to the new species

**Table:** 

1a	Leaves keeled; leaflet insertion at an acute angle to the rachis	**2**
2a	Leaflets strongly imbricate up to two thirds of the leaf length	***D. califanoi***
2b	Leaflets non-imbricate or very slightly imbricate up to one third of the leaf length	**3**
3a	Leaflets with 1–5 serrations distally; silvery pubescence at emergence and senescence	***D. argenteum***
3b	Leaflets with 2–3 serrations or entire distally; glabrous at emergence and maturity	**4**
4a	Petiole base lanate at maturity; microsporophyll apex straight	***D. purpusii***
4b	Petiole base glabrous at maturity; microsporophyll apex curved	***D. planifolium***
1b	Leaves plane; leaflets inserted at right to slightly acute angles along rachis	**5**
5a	Leaflets imbricate	**6**
6a	Margin not acroscopically curved, with 1–2 serrations on the margin; new leaves golden	***D. stevensonii***
6b	Margin acroscopically curved, with 3–6 marginal serrations; new leaves light green	. ***D. nuusaviorum* sp. nov**.
5b	Leaflets non-imbricate	**7**
7a	Leaflets linear, ≤ 0.6 cm wide	***D. caputoi***
7b	Leaflets linear-lanceolate, > 0.6 cm wide	**8**
8a	Leaflets with 3–4 serrations to entire distally	***D. holmgrenii***
8b	Leaflets with 1–2 serrations or entire distally	**9**
9a	Leaflets slightly falcate in the apical portion	***D. salas-moralesiae***
9b	Leaflets non-falcate in the apical portion	***D. oaxacensis***

## Discussion

Historically, qualitative morphological characters of vegetative structures have been relevant for species diagnosability in *Dioon* (De Luca et al. 1982; Vovides et al. 2007). Some species are nearly indistinguishable from their congeners based on quantitative vegetative characters, yet strikingly different in both qualitative and quantitative reproductive traits. In particular, ovulate strobilus morphology often provides a unique combination of diagnostic character states (Table 1). However, such traits cannot be used to identify pollen-bearing or purely vegetative plants. Recent studies have demonstrated the informativeness of combining molecular data with reproductive and vegetative morphology (Nicolalde-Morejón et al. 2009; Gutiérrez-Ortega et al. 2020b, 2021).

Our field observations indicate that *Dioon nuusaviorum* exhibits a reproductive phenological pattern in which ovulate individuals within a population are simultaneously in receptive and disintegration phenophases. Phenological data have contributed to taxonomic hypotheses and circumscriptions, as well as recently provided evidence on evolution and potential hybridization in cycads (Martínez-Domínguez et al. 2022a, 2022b, 2024b). However, the vegetative and reproductive cycles of *Dioon* have been poorly explored and recorded (Mora et al. 2013). This is aggravated by the fact that ovulate strobili in *Dioon* may require up to two years to develop from emergence to seed release (Norstog and Nicholls 1997).

*Dioon nuusaviorum* has been likely overlooked due to the pattern of qualitative, discrete variation found in the populations of southeast Guerrero (Figs 1, 4, Tables 1, 2). This taxon is geographically close to *D. stevensonii*, which occurs from central to northwest Guerrero into southeastern Michoacán. However, the two species are not sympatric. Our analyses were based on a broad sampling effort consistent with recent recommendations emphasizing the inclusion of neighboring populations when evaluating intra- and interpopulation variations, reproductive phenology, and potential hybridization (Gutiérrez-Ortega et al. 2018b; Martínez-Domínguez et al. 2024a, 2024b).

Phylogenetic relationships among *Dioon* species have been investigated by several authors over more than three decades (Moretti et al. 1993; Vovides et al. 2007; González et al. 2008; Dorsey et al. 2018; Gutiérrez-Ortega et al. 2018a, 2018b). The major lineages recovered in those phylogenetic and phylogeographic studies, using different approaches and methods, were highly consistent (Dorsey et al. 2018; Gutiérrez-Ortega et al. 2018a, 2018b). These results have suggested that geographic isolation has promoted reproductive isolation (Gutiérrez-Ortega et al. 2018a), leading to lineages that correspond closely to species distributions. In this context, the proposed new species may represent the sister species of *D. stevensonii*; however, molecular phylogenetic data are needed to test this hypothesis.

Recent studies on the diversity and genetic structure of *Dioon holmgrenii* populations in Oaxaca, carried out for conservation purposes, revealed low genetic diversity and limited gene flow, with only moderate fragmentation and a substantial portion of shared alleles among populations (Velasco-García et al. 2021; Dorsey et al. 2024). One population in northern Oaxaca showed little difference in leaf morphology, suggesting a degree of divergence, but may also be the result of anthropogenic activities (Dorsey et al. 2024). In contrast to vegetative characters in cycad species that can have high variation related to local ecological adaptations (Limón et al. 2016), reproductive characters exhibit far less random variation or size alterations resulting from temporary environmental issues, probably because they are directly linked to reproduction and therefore might contribute to the reproductive fitness of the species (Martínez-Domínguez et al. 2022b).

With the description of *Dioon nuusaviorum*, the total number of known *Dioon* species would increase to 19, with 18 of these taxa occurring in Mexico (Calonje et al. 2025). Given its restricted distribution and the high incidence of fires and other anthropogenic pressures in this area of the Sierra Madre del Sur, we propose a preliminary IUCN category of Endangered for this species. Further studies of genetic diversity, demography and phenology are needed to better evaluate the conservation status of *Dioon* species, and to test hypotheses regarding vicariant speciation within the genus.

## Supplementary Material

XML Treatment for Dioon nuusaviorum

## Additional information

### Use of AI

No use of AI was reported.

### Author ORCIDs

Lilí Martínez-Domínguez https://orcid.org/0000-0003-1158-1501

Fernando Nicolalde-Morejón https://orcid.org/0000-0003-1423-7474

Dennis Wm. Stevenson https://orcid.org/0000-0002-2986-7076

Francisco Vergara-Silva https://orcid.org/0000-0001-5024-268X

### Data availability

All of the data that support the findings of this study are available in the main text or Supplementary Information.

## Supplementary materials

Supplementary material 1 Supplementary information Data type docx https://binary.pensoft.net/file/1631308This dataset is made available under the Open Database License (http://opendatacommons.org/licenses/odbl/1.0/). The Open Database License (ODbL) is a license agreement intended to allow users to freely share, modify, and use this Dataset while maintaining this same freedom for others, provided that the original source and author(s) are credited. Lilí Martínez-Domínguez, Fernando Nicolalde-Morejón
