# Supplementary material for: A striking new species of Dioon (Zamiaceae) from pine and pine-oak forest of Guerrero, Mexico
Source: PhytoKeys. 2026 May 11;274:229–45. doi: 10.3897/phytokeys.274.173907 (PMC13184629; doi:10.3897/phytokeys.274.173907)

**Table S1.** Populations for quantitative morphological variation.

| *Dioon* species | Locality | Elevation |
| --- | --- | --- |
| *Dioon* sp. nov. | Tlacoachistlahuaca, Near Rancho Viejo, | 1190 |
| *Dioon* sp. nov. | Tlacoachistlahuaca, La Trinidad | 1030 |
| *Dioon* sp. nov. | Near Rancho Viejo | 1050 |
| *D. stevensonii* | Zirándaro, La ciénega | 1050 |
| *D. stevensonii* | Arteaga, El Higueral | 675 |
| *D. holmgrenii* | Santa María Colotepec, Valdeflores hacia San Bartolomé Loxicha | 800 |
| *D. holmgrenii* | San Gabriel Mixtepec | 500 |
| *D. holmgrenii* | San Bartolomé Loxicha, Rancho el Limón | 970 |

**Table S2**. Qualitative morphological characters and their respective character states (vegetative and reproductive).

| Character | Character state |
| --- | --- |
| Cataphylls apex shape | (0) acuminate; (1) acute; (2) rounded |
| Trichomes on cataphylls (vestiture) | (0) completely tomentose; (1) partially tomentose; (2) pubescence |
| Cataphylls shape | (0) triangular; (1) long triangular |
| Trichomes color on leaf at emergence | (0) golden; (1) light brown; (2) brown |
| Leaf color at emergence | (0) light-green; (1) Golden brown |
| Leaf orientation | (0) ascending; (1) descending |
| Leaflet insertion on the rachis | (0) keeled; (1) plane |
| Arrangement of leaflets on the rachis | (0) opposite; (1) sub-opposite |
| Leaflet shape | (0) linear; (1) lanceolate |
| Leaflet direction | (0) falcate; (1) not falcate |
| Leaflet imbrication | (0) imbricate; (1) not imbricate |
| Leaflet direction on abaxial side | (0) curved downward; (1) straight |
| Direction leaflet apex | (0) reflexed; (1) not reflexed |
| Denticles appearance | (0) fine; (1) strong |
| Leaflet position on rachis (angle) | (0) right; (1) acute |
| Apex shape of microsporophylls | (0) acuminate; (1) acute |
| Sterile portion reflex | (0) present; (1) absent |
| Sterile portion apex | (0) pungent; (1) rounded; (2) slight pungent |
| Indument on megasporophylls at maturity | (0) tomentose; (1) pubescence; (2) pubescence at base |
| Basal scales megasporophylls color | (0) light green; (1) green; (2) yellowish green; (3) dark green |
| Apex shape of basal scales megasporophylls | (0) acute; (1) apiculate |
| Apex shape of megasporophylls | (0) apiculate; (1) acuminate; (2) acute |
| Apex shape of microsporophylls | (0) acuminate; (1) acute |
| Seeds shape | (0) spherical; (1) ovoid |

**Table S3**. Quantitative morphological characters (vegetative and reproductive). In bold are characters used in morphometric analysis.

| Character | Abbreviation |
| --- | --- |
| Length of cataphylls | - |
| Width of cataphylls | - |
| **Number of leaves** | **NL** |
| **Pairs of leaflets** | **PLe** |
| **Length of petiole** | **PL** |
| **Length of rachis** | **RL** |
| **Total length of leaves** | **TL** |
| **Length of basal leaflet** | **LBL** |
| **Width of basal leaflets** | **WBL** |
| Width of basal leaflet articulation | - |
| **Distance between basal leaflets** | **DbBF** |
| **Length of median leaflets** | **LML** |
| **Width of median leaflets** | **WML** |
| Width of median leaflet articulation | - |
| Number of veins in median leaflet | - |
| **Distance between median leaflets** | **DbML** |
| **Length of apical leaflet** | **LAL** |
| **Width of apical leaflet** | **WAL** |
| Width of apical leaflet articulation | - |
| **Distance between of apical leaflets** | **DbAL** |
| **Number of denticles on leaflet** | **DN** |
| **Length of denticles on leaflet** | **DL** |
| Length of pollen strobilus peduncle | - |
| Diameter of pollen strobilus peduncle | - |
| Length of pollen strobilus (fertile portion) | - |
| Diameter of pollen strobilus (fertile portion) | - |
| Length of microsporophylls | - |
| Width of microsporophylls | - |
| Number of synangia per microsporophylls | - |
| Length of ovulate strobilus (fertile portion) | - |
| Diameter of ovulate strobilus (fertile portion) | - |
| Length of ovulate strobilus peduncle | - |
| Diameter of ovulate strobilus peduncle | - |
| Length of megasporophylls | - |
| Width of megasporophylls at base | - |
| Length of seed | - |
| Diameter of seed | - |

**Figure S1.** Morphometric variation among three examined species using box plot. A. Length of apical leaflet; B. Distance between median leaflets; C. Length of median leaflets; D. Width of median leaflets; E. Length of denticles on leaflet; F. Number of leaves.


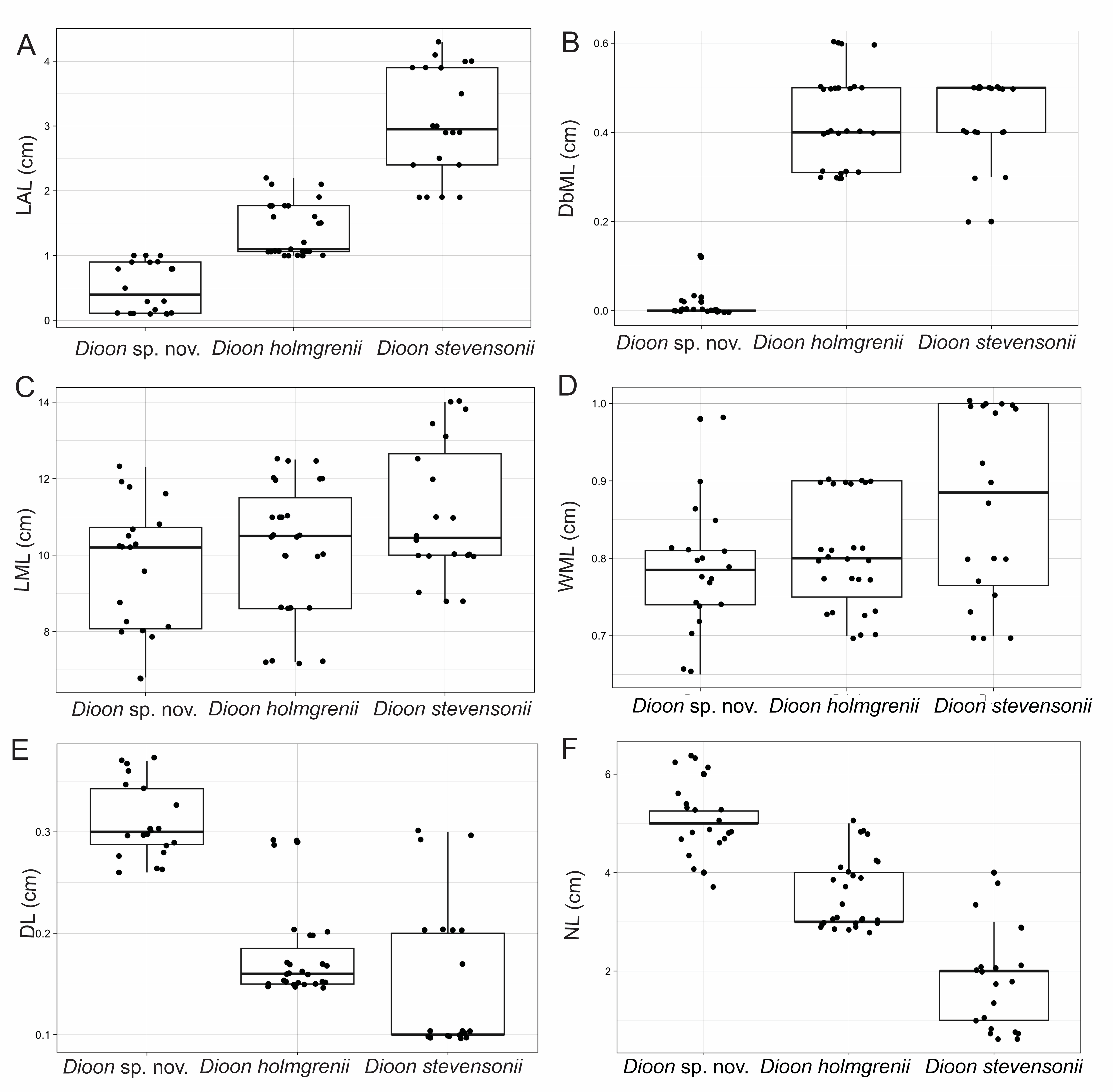


**Table S4.** Results from Tuckey test.

| Quantitative character | Mean Sq | F value | Pr (>F) |
| --- | --- | --- | --- |
| NL | 1716.5 | 19.57 | 2.33e-07 |
| PLe | 25617 | 80.48 | <2e-16 |
| PL | 1240.4 | 80.2 | <2e-16 |
| RL | 5116 | 11.99 | 3.79e-05 |
| TL | 7710 | 15.02 | 4.47e-06 |
| LML | 11.212 | 3.735 | 0.00292 |
| WML | 0.04186 | 5.082 | 0.00894 |
| DbML | 1.257 | 180.3 | <2e-16 |
| DN | 54.61 | 93.16 | <2e-16 |
| DL | 0.14783 | 51.92 | 3.99e-14 |
| LBL | 19.515 | 40.18 | 4.59e-12 |
| WBL | 0.07547 | 7.921 | 0.000844 |
| DbBF | 1.8807 | 34.26 | 7.67e-11 |
| LAL | 33.98 | 106 | <2e-16 |
| WAL | 0.06563 | 32.57 | 1.76e-10 |
| DbAL | 0.3732 | 2.906 | 0.0619 |

**Figure S2.** PCA plot that summarizes the variation among three species.


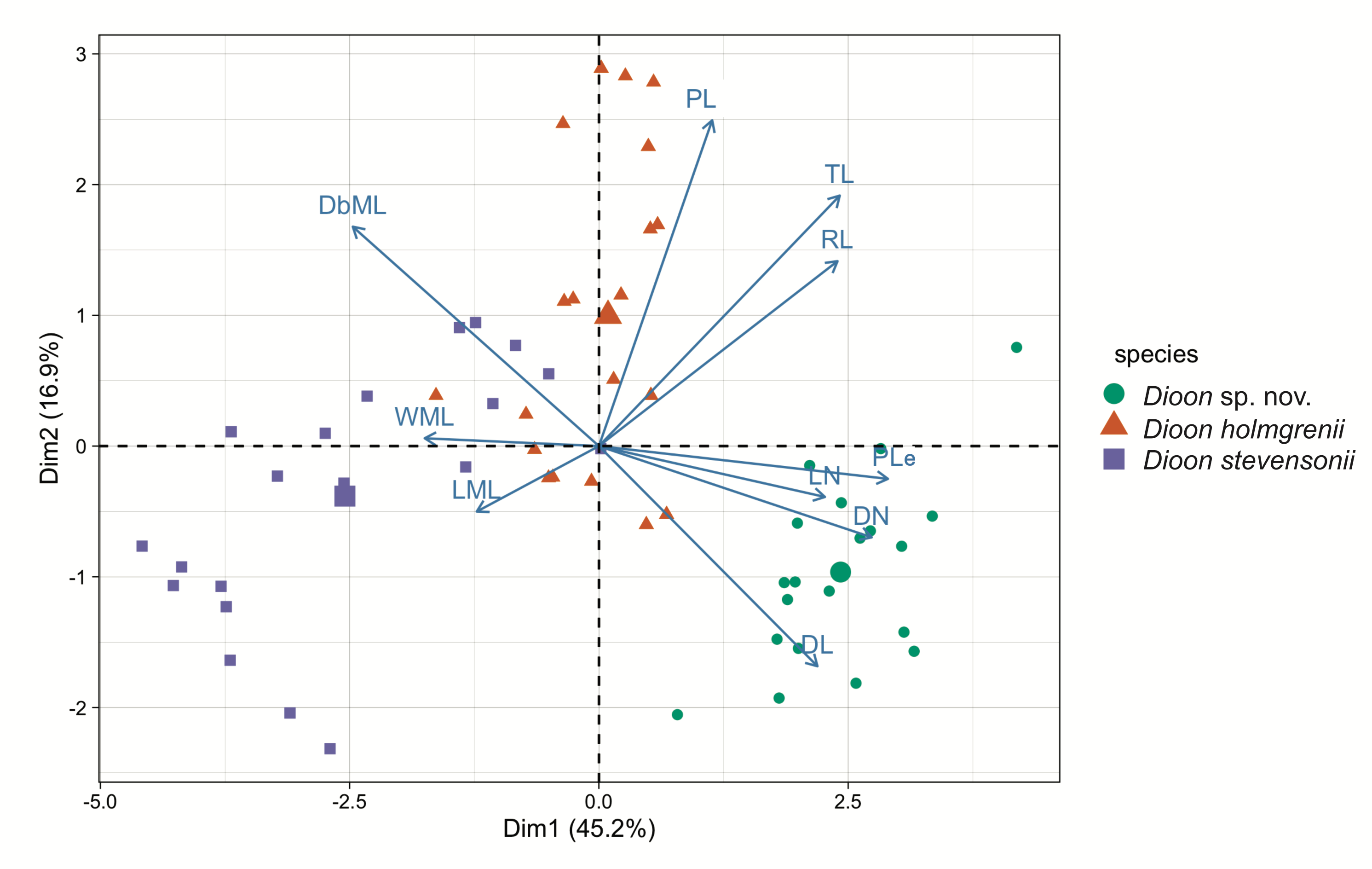

Supplement: Supplementary material 1 — Supplementary information [file phytokeys-274-229_article-173907__-s001.docx]
